# Parkinson's Disease: Unanticipated Sequela of an Attempted Suicide

**DOI:** 10.7759/cureus.9409

**Published:** 2020-07-26

**Authors:** Jonathan T Grossman, Asia Filatov, Thomas Hammond

**Affiliations:** 1 Neurology, Charles E. Schmidt College of Medicine, Florida Atlantic University/Marcus Neuroscience Institute, Boca Raton, USA; 2 Neurology, Charles E. Schmidt College of Medicine, Florida Atlantic University, Boca Raton, USA; 3 Neurology, Marcus Neuroscience Institute, Boca Raton, USA

**Keywords:** parkinson' s disease, pesticide, mptp, kratom, basal ganglia

## Abstract

Studies exploring the association between pesticide exposure and Parkinson's disease (PD) are largely limited to rural populations with occupational exposure. We could find no literature regarding PD occurring after ingestion of a pesticide. We present a case from our clinic of a man who developed PD following ingestion of a liquid pesticide during a suicide attempt. PD was diagnosed, and the patient’s symptoms improved following initiation of carbidopa/levodopa. This case illustrates the potential role of ingested pesticide exposure in provoking and accelerating the manifestations of PD.

## Introduction

During the late 1950s and early 1960s, eloquent medical research by Arvid Caarlson (Nobel Prize 2000) and Oleh Hornikiewicz led to the discovery of dopamine depletion in the striatum and dopaminergic cell loss in the substantia nigra (SN) in patients with Parkinson's disease (PD) [1]. Subsequent clinical trials in the 1960s gave levodopa to patients with PD. The administered levodopa produced significant improvement in the core clinical PD features of tremor, bradykinesia, rigidity, and postural instability. Levodopa therapy remains our most effective therapy for PD. The etiology of PD and the cause of cell loss remained unclear. In 1982 arose a designer drug culture in Northern California where synthetic chemicals were being developed for recreational use. A compound was developed in a garage laboratory akin to “synthetic heroin” 1-methyl-4-phenyl-1,2,3,6, tetrahydropyridine (MPTP) [2]. This compound was used by seven individuals who presented with the acute onset of clinically advanced PD. The work by J. W. Langston found that MPTP caused severe cell loss in the pigmented (dopaminergic) neurons of the SN producing the PD symptoms which were responsive to levodopa [2]. This discovery led to animal models of PD which accelerated PD research [3]. Furthermore, an MPTP metabolite was chemically similar to paraquat (a commonly used herbicide) and this fact led to studies of PD and pesticide use in agriculture [2]. An increase in prevalence of PD was found to be associated with pesticide exposure. In 1999, a study of World War II (WWII) veterans who were twins examined PD [3]. Over the age of 50 years, if one twin developed PD, the monozygotic and dizygotic twin developed PD at the same 10% rate. However, below age 50 years the monozygotic twin always developed PD (100%), while the 10% rate occurred in the dizygotic twin. While genetic predisposition appears to be the most significant risk factor for developing PD below age 50 years, above that age genetic predisposition interacts with environmental or other factors. Hence, the expression “genetics loads the gun and the environment pulls the trigger” [4]. Epidemiological research has suggested that two commercial agrochemicals, paraquat and maneb, are apparent triggers for PD [5]. Paraquat is typically used during the growing process and maneb is applied following harvesting to prevent spoiling. Because the contribution of pesticides to the evolution of PD has been presumed to be dependent on both dose and frequency of exposure, a focus on farming/rural communities has emerged. Presently, we are unaware of literature regarding the ingestion of pesticide and its potential contribution to developing PD. In this case report, we describe the evolution of PD following an attempted suicide via the intentional ingestion of pesticides. 

## Case presentation

**Figure 1:**
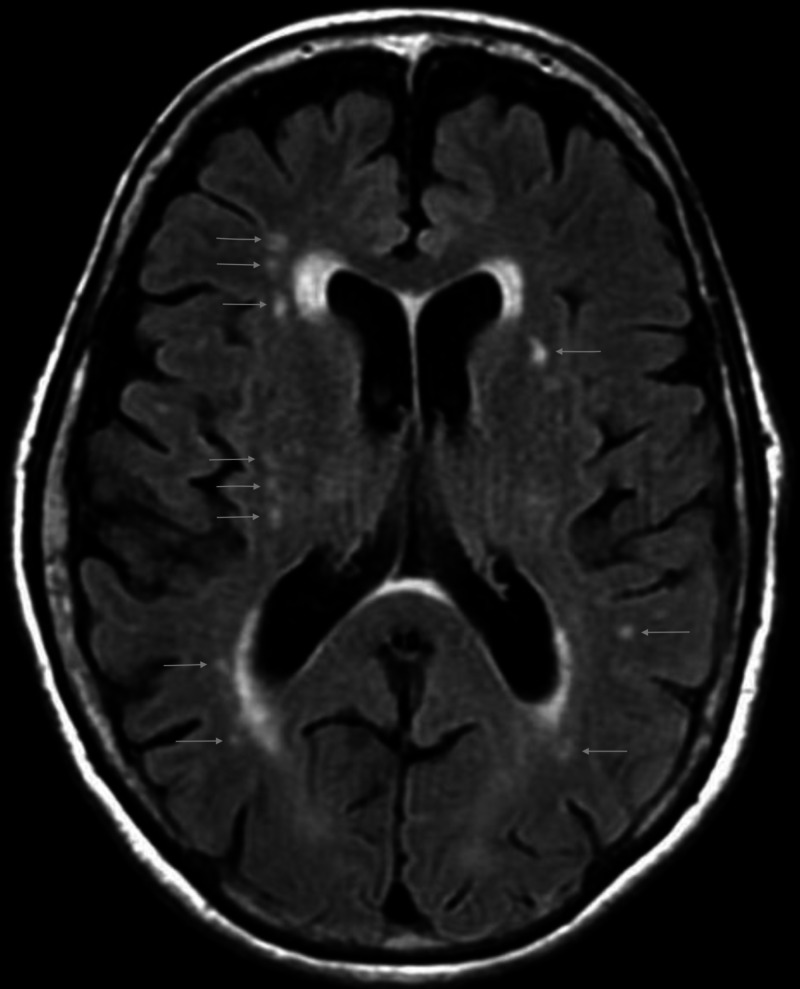
Multiple T2 hyperintense foci can be seen in the white matter on axial FLAIR consistent with chronic microvascular ischemic changes. FLAIR, fluid-attenuated inversion recovery

A 65-year-old male with a past medical history significant for major depressive disorder and generalized anxiety disorder attempted suicide via intentional ingestion of two ounces of Termidor, a liquid insecticide. He was hospitalized at that time following the abrupt onset of ataxia, confusion, and seizures. Thereafter, he spent a brief time in a behavioral health facility. He had noticed an occasional tremor of the left hand before his suicide attempt. During the two years following hospitalization for pesticide ingestion, he developed slowness of movements with particularly diminished arm swing (left > right) and diminished facial expression and eye blinking. He developed softened speech and micrographic handwriting. He developed difficulty swimming which he had enjoyed as exercise. He developed a prominent resting hand tremor (left > right). All of these PD features showed marked improvement following the diagnosis of PD and initiation of carbidopa/levodopa in November 2018. Prior to this suicide attempt, he had been using alprazolam for many years to manage his generalized anxiety. He had attended a detoxification program to wean from alprazolam and was successfully transitioned from alprazolam to a combination of olanzapine (briefly used), bupropion, and clonazepam (also briefly used) by a psychiatrist. He underwent electroencephalogram (EEG) at that time which was unremarkable. Neuroimaging at that time included an unremarkable CT brain and an MRI brain (Figure 1), with white matter chronic microvascular ischemic changes without any appreciable signal changes in the basal ganglia. 

For his anxiety, he has continued taking one teaspoon of “Kratom” (an herbal opioid agonist with some stimulant effect) dissolved in water, approximately four times per day. The patient feels this compound has helped both his anxiety and sleep difficulties. He has tried sertraline for his depression and buspirone for his anxiety, both of which found to be ineffective. He continues to do well using carbidopa/levodopa for his PD symptoms. 

## Discussion

PD is a progressive neurodegenerative disease that affects movement. The cardinal clinical features begin insidiously with resting tremors on one side which eventually spread to both sides. Akinesia is the most significant PD feature with slowing of bodily movement, including diminished facial expression, eye blinking, and arm swing. A forward stooped posture and shuffling, narrow-based gait are additional hallmark clinical features. Impaired postural stability occurs as the disease progresses. The pathologic features are progressive loss of dopaminergic neurons in the SN with intracellular inclusions called Lewy bodies. The Lewy bodies contain a protein alpha synuclein. While specific genes have been identified that play a role in developing PD (LRRK2, PARK7, PINK1, PRKN, SNCA), pesticide exposure has been implicated as a possible precipitant of disease [6]. The current patient had onset of PD following the intentional ingestion of a liquid pesticide called Termidor. This agent is a commonly used termiticide that contains fipronil (5-amino-1-[2,6-dichloro-4-(trimethyl ethyl)sulfinyl]-1H-pyrazole-3-carbonitrile). First discovered in 1987, this broadly used insecticide belongs to the phenylpyrazole family and has been approved for use in the United States since 1996 [7]. Aside from its agricultural use in soil treatment and crop protection, fipronil is also available for use as the topical applicant (aka Frontline) for dogs and cats to mitigate flea, tick, and mite infestations. Fipronil disrupts GABA- and glutamate-gated chloride channels in the central nervous system of insects. This results in blocking inhibitory neurotransmission that leads to hyperexcitability (at lower doses) followed by paralysis and death (at higher doses). It is presumed that these adverse effects are limited to insects due to its high affinity for specific receptor subtypes including GABA_A_; the binding affinity for this receptor is far greater in arthropods than in humans. This may account for its reported low risk for acute toxicity. Furthermore, glutamate-gated chloride channels have only been observed in invertebrates. In the case of humans, ingestion appears to be followed by rapid and complete absorption in the GI tract with extensive metabolism to the sulfone derivative followed by significant enterohepatic recirculation [7]. Neurotoxicity following ingestion is better understood in insects than in mammals but signs of neurotoxicity following ingestion by laboratory animals have been observed. These signs included gait abnormalities, tremors, convulsions, and postural changes including stooped posture [7]. Ingestion by humans was either inadvertent or secondary to a suicide attempt. Reported signs/symptoms following human ingestion included headache, generalized or tonic-clonic seizures (our patient had seizures during the initial hospitalization), paresthesia, pneumonia, and death. Significant associations between PD incidence and pesticide exposure (e.g. alachlor, broxomy) exist, and exposure to such chemicals is thought to interrupt the functions of cells in a manner that parallels the impacts of mutations that are known to cause PD [8]. Individuals exposed to a variety of agrochemicals have a near 250% increased risk of developing PD when compared to control populations [9]. While studies regarding the cumulative effect of exposure to agrochemicals (e.g. inhalation, cutaneous absorption) are well documented, there remain no reports to date that explore the adverse effects or sequelae that follow inadvertent or intentional ingestion of insecticides [10]. There does appear to be a propensity for the development of PD following exposure to agrochemicals in patients with an underlying genetic predisposition. It is important to note that before ingestion, aside from his chronic anxiety and depression our patient did describe an occasional resting left-handed tremor. This may have represented an early sign of PD. However, the rapid progression of PD over the 1.5 to 2 years following ingestion of Termidor (specifically, fipronil) suggests an accelerated course of PD. In developing nations, the substance most frequently used in an attempted suicide is an agricultural pesticide [10]. Still, no reports regarding PD following such suicide attempts were found. 

## Conclusions

The association between exposure to agrochemicals and their influence on the development of PD continues to be explored. This case report presents a unique exposure history with a solitary, intentional ingestion of pesticide. Most of the literature currently published describes a population with known frequency or dose-dependent exposure, e.g. farmers, agricultural workers, or more simply those residing in rural settings. Notably, our patient reported an occasional resting tremor of the left hand in the year prior to ingestion. This fact suggests that his PD was in its early evolution before pesticide ingestion and that this exposure served as a potentiating factor accelerating the progression of disease. 
